# Rare Disease Focused Antenatal Education and Diagnosis Support: Two Case Studies of Epidermolysis Bullosa Simplex

**DOI:** 10.1186/s13023-024-03397-2

**Published:** 2024-10-11

**Authors:** Rebecca Saad, Ryan Pysar, Alaxandra Blackwell

**Affiliations:** 1 National Epidermolysis Bullosa Dressing Scheme. Program funded by the Federal Department of Health and Aged Care, and administered by Independence Australia, Melbourne, Australia; 2 Department of Dermatology, Sydney Children’s Hospital, Randwick, NSW Australia; 3 Centre for Clinical Genetics, Sydney Children’s Hospital, Randwick, NSW Australia; 4 Department of Clinical Genetics, Children’s Hospital at Westmead, Westmead, Australia; 5 Discipline of Paediatrics and Child Health, School of Clinical Medicine, Faculty of Medicine and Health, University of New South Wales, Sydney, NSW Australia

**Keywords:** Epidermolysis Bullosa, Antenatal, Newborn, Rare disease, Referral, Diagnosis, Support

## Abstract

Many people living with a rare disease (RD) face challenges accessing timely diagnosis and disease-specific specialist care. Early health-care challenges for people living with Epidermolysis Bullosa (EB), a rare genetic disease affecting 1:20,000 individuals, can begin in the antenatal period.

People living with EB in Australia have access to a government funded disease specific antenatal education and support program through the National Epidermolysis Bullosa Dressing scheme (NEBDS). This article discusses two births involving families living with EB Simplex (EBS) in regional Australia. The education and support structures implemented by the NEBDS and clinical teams are discussed in line with the Australian National Strategic Action Plan for rare diseases, and includes access to genetic diagnosis, EB education, and complex care coordination.

## Introduction

Epidermolysis Bullosa (EB) is classified as a rare disease with an incidence of 1:20,000 [1] characterised by skin fragility where mechanical friction to the skin causes wounds or blisters [1, 2]. Medical monitoring and screening protocols recommended through pregnancy and childbirth can pose substantial health risks when a diagnosis of EB is suspected [2–5]. Mechanical friction encountered during handling and medical monitoring can contribute to increased blistering and wound development for the mother and/or newborn while in hospital [2–5]. This article will explore how two cases of EB antenatal support addressed the challenges of EB rare disease (RD) diagnosis and support.

### Regional rare disease antenatal and diagnosis support

Individuals living with a RD frequently encounter challenges accessing early and accurate diagnosis as well as access to antenatal and reproductive support [2, 6–8]. Access to specialist RD care for regional and rural families requires travel to major cities, time away from family, interruptions to work, and increased financial burden to the family [9–11]. These factors exacerbate health disparities when compared with Australians residing in urban areas resulting in poorer health outcomes [6, 9]. The Australian National Strategic Action Plan for Rare Diseases [6] identifies ‘Care and Support’ that includes access to timely and accurate diagnosis, facilitation of reproductive confidence, and enabling equitable access to care as being critical elements of RD care.

### Antenatal education

Australians living with EB that have access to Medicare (Australia’s universal health insurance scheme) can join the National EB Dressing Scheme (NEBDS), funded by the Federal Governments’ Department of Health and Aged care. The NEBDS provides heavily subsidised wound care products, and EB education programs for schools and hospitals to support the clinical wound care of people living with EB.

The NEBDS antenatal program, incorporates RD management principles outlined in the National Strategic Action plan for Rare diseases [6] with EB best practice guidelines for diagnosis [1], pregnancy care [2], and wound care [3]. The NEBDS antenatal program was developed by the NEBDS nurse, and includes the following:

- Acknowledgement of the patient as a disease expert and inclusion in health care planning decisions.
- EB subtype specific education and support for patient and local health care providers.
- Referral support for access to early genetic counselling and genomic testing.
- Access to subtype specific wound care including low adherence products and EB trained wound care staff.
- Implementation of preventative care for all monitoring and screening procedures.

An Antenatal referral and education form [12] facilitates complex care coordination and communication between all stakeholders and is used to communicate and summarise the education and support provided to families and Health Care Professionals (HCPs). Individuals or HCPs can refer themselves or their client to the NEBDS for antenatal support.

### Genetic counselling

EB subtypes can display autosomal dominant or recessive inheritance patterns. EB Simplex (EBS) can occur through either mode of inheritance [1, 13]. An affected parent with a dominant form of EBS has a 50% chance of their offspring inheriting the condition [1, 13]. Affected parents may access genetic counselling when planning a pregnancy or during the pregnancy as anxieties about recurrence and reproductive decision-making are common. Genetic counselling can facilitate decision-making about options for investigating genetic diagnosis, whether prenatally or postnatally [1] and should be offered to individuals living with EB.

## Case 1

### Maternal antenatal and newborn EB support

Mother 1 (M1) has a genetic diagnosis of EB Simplex (EBS) due to a pathogenic variant in *KRT14*. She had family support with her EB, lived in a regional area 2-hrs drive from the closest EB specialist team. Her local birthing hospital did not have access to complex newborn wound support. Specialised EB care was required during M1’s pregnancy and for her newborn.

### Genetic counselling and diagnosis

M1 was referred to a clinical genetics team at 6- weeks gestation. A telehealth appointment was arranged for M1 with a clinical geneticist present to discuss prenatal diagnosis via chorionic villus sampling (CVS) and reproductive decision-making. After a follow up consultation with her local genetics service, M1 elected to undergo a CVS at 12-weeks. Expedited molecular clarification of EBS in M1 confirmed the pathogenic *KRT14* variant. The CVS confirmed that the pathogenic *KRT14* variant was inherited in the newborn, and therefore established a genetic diagnosis of EBS.

### Management

M1 self-referred to the NEBDS program and provided written consent for collaborative communication between HCPs. The NEBDS nurse, M1, and the obstetrician met virtually (due to covid restrictions) to discuss expectations of care, service provisions and staff capabilities at the local regional birthing hospital. The reduced capacity for complex newborn wound care was identified and M1s care was transferred antenatally to a tertiary care hospital close to EB specialists.

The NEBDS nurse facilitated communication between the EB specialist team, the local obstetrician, the clinical genetics service, and the tertiary care hospital. The NEBDS nurse provided EB education for the Maternal fetal medicine unit, midwives, medical staff in the delivery suit, as well as the neonatal intensive care unit.

EBS-specific wound care products and resources were stored in an ‘EB Box’ in preparation for the birth. Items not held by the birthing hospital were ordered through the NEBDS. The birthing hospital created a neonatal high risk birth plan using EB resources and educational content provided by the NEBDS nurse sourcing clinical best practice guidelines [2, 3].

### Outcome

The educational resources, management plan, and EB Box were available throughout the birth including preventative padding and lubrication and use of low adherence wound care items. No new blisters or wounds occurred for M1 during monitoring or handling throughout her birth or postnatal recovery.

Newborn 1 (N1) was born vaginally and management plan prevention strategies were implemented to reduce skin friction from monitoring. Blistering to their hands caused by sucking during gestation were present at birth. Antenatal blister management training, previously provided by the NEBDS nurse, allowed staff to promptly treat all blisters with early draining and appropriate padding to prevent further blisters or wounds.

The NEBDS nurse advised the NEBDs program of a pending new application for wound care items. Early access to EB wound care and transfer to the EB specialist team contributed to the newborn experiencing minimal new blisters, and a hospital discharge home on day 5 of life.

## Case 2

### Birth and newborn support for suspected EB

Mother 2 (M2) has no EB diagnosis, was being supported for the pregnancy and birth of her 3rd child. M2’s husband and two children have been diagnosed with EBS. The family reside 7-hours drive from the closest EB specialist team. Their local birthing hospital was a level 5 teaching hospital able to support complex births and wound care.

### Genetic counselling and diagnosis

M2 self-referred to the genetic counsellor at 12-weeks gestation. After genetic counselling that included discussion about timing of genetic testing for the pregnancy, the family elected to collect postnatal cord blood for genetic testing in the newborn. The plan to test cord blood was communicated with the birthing team and request forms for cord blood collection for genetic testing were made available prior to the birth.

### Management

M2 self-referred to the NEBDS program and provided written consent for communication between public and private providers. The local obstetrician and paediatrician developed a high-risk birthing and neonatal management plan using information provided by the NEBDS nurse, sourcing clinical best practice guidelines [2, 14]. The NEBDS nurse hosted two online EB education sessions for a total of 20 hospital staff at the birthing hospital of M2. The parents’ EB knowledge and skills were promoted by the NEBDS nurse, recognised by the birthing hospital staff, and included in the birthing plan.

An EB box (as described in Case 1) was created. It was agreed that birth and postnatal management would occur at the local birthing hospital, and only severe wounds or clinical complications of the newborn postnatally would lead to consideration of air transfer to the EB specialist team.

### Outcome

The EB box and management plan accompanied M2 through labour, operating theatres, newborn care, and postnatal ward. The EB management plan was implemented at birth to minimise blister development should the newborn be affected with EBS and promoted inclusion of the father for handling during newborn monitoring procedures.

Newborn 2 (N2) experienced no signs of EB in the first 48-hours. After a caesarean section birth (for maternal medical reasons), cord blood was collected as planned and sent for genetic testing. Local HCPs initiated contact with the EB specialist team on day 3 of life when N2 developed blisters on fingers and slight marking and redness on the skin after handling. Care was managed locally with telehealth and phone support from the EB team until discharge on day 6.

The clinical diagnosis of EBS, given on day 3, was confirmed at eight weeks, when the genetic cord blood identified the familial EBS genetic variant. The genetic counsellor discussed results and provided emotional support via telehealth.

## Discussion

The known challenges of patients living with RD were prioritised during planning and delivery of the NEBDS antenatal program specifically; EB specific education, collaborative communication between families, local HCP and EB specialists during care planning. This framework was integral to patient empowerment, safety, and risk reduction by ensuring HCP education and hospital resources are in place prior to delivery.

The common RD issues of ‘reproductive empowerment’ and ‘equity of access to health care’ were addressed through the antenatal program and promoted the following:

- Establishing early collaboration and communication between all stakeholders including recognising the family as an expert.
- Supporting choice timing of genetic diagnosis and access to clinical genetics services (if not already linked).
- Establishing access to EB wound care for the mother and/or newborn to ensure access to the EB box containing information and products for preventative cares such as extra padding, lubrication, and use of low adherent tapes and dressings during hospital procedures.
- Providing education and educational resources to the birthing hospital.
- Providing educational support until the birth when the case is handed over to the EB specialist provider should any specific clinical care of the mother of newborn be required.
- The NEBDS role is primarily education, providing resources and support to enable to the clinical team to establish appropriate patient specific treatment plans.

## Conclusion

Through the support of early comprehensive antenatal care, referral for diagnosis, and disease specific management [1, 6] HCPs can promote reproductive confidence [2, 6] for families living with a RD. Reproductive health equity can be addressed when HCPs integrate mental, social, and emotional support through a rare disease framework [6, 14, 15]. Early referral to a specialist provider can reduce the risk of new wounds for the newborn suspected of EB. Preventative handling that decreases friction and early use of low adherent wound care for newborns with EB are crucial if further complications of wound severity, pain, and infection are to be avoided [3, 5, 16].

## Data Availability

All data necessary to support the case studies have been included in the manuscript. Individual consent from families has been provided to the journal and is not available for public review to protect patient privacy.

## Declarations

### De-identification of subjects

Every effort has been made to de-identify patients and their cases. Patients, clinicians, and clinical sites have not been named.

## Abbreviations

CVS: Chorionic Villus Sampling
EB: Epidermolysis Bullosa
EBS: Epidermolysis Bullosa Simplex
M1: Mother 1
M2: Mother 2
N1: Newborn 1
N2: Newborn 2
MMI: Mother of M1
NEBDS: National Epidermolysis Bullosa Dressing Scheme
NSW: New South Wales
RD: Rare Disease

## References

[1] Has C, Liu L, Bolling M, Charlesworth A, El Hachem M, Escámez M, et al. Clinical practice guidelines for laboratory diagnosis of epidermolysis bullosa. Br J Dermatol. 2020;182(3):574–92. 31090061 10.1111/bjd.18128 PMC7064925

[2] Greenblatt DT, Pillay E, Snelson K, Saad R, Torres Pradilla M, Widhiati S, et al. Recommendations on pregnancy, childbirth and aftercare in epidermolysis bullosa: a consensus-based guideline. Br J Dermatol. 2022;186(4):620–32. 34687549 10.1111/bjd.20809 PMC9298908

[3] Has C, El Hachem M, Bučková H, Fischer P, Friedová M, Greco C, et al. Practical management of epidermolysis bullosa: consensus clinical position statement from the European Reference Network for Rare skin diseases. J Eur Acad Dermatol Venereol. 2021;35(12):2349–60. 34545960 10.1111/jdv.17629

[4] Denyer J, Pillay E, Clapham J. Best practice guidelines for skin and wound care in Epidermolysis Bullosa. Int Consensus Wounds Int. 2017.

[5] Saad S, Duipmans J, Yerlett N, Plevey K, McCuaig C, Woolfe W, et al. Neonatal epidermolysis bullosa: a clinical practice guideline. Br J Dermatol. 2024;ljae006. 10.1093/bjd/ljae006. Accessed 16 April 2024. 10.1093/bjd/ljae006 38175636

[6] Australian Government Department of Health and Aged Care. National Strategic Action Plan for rare diseases. In: Health Do, editor. 1 ed2020. https://www.health.gov.au/resources/publications/national-strategic-action-plan-for-rare-diseases. Accessed 16 April 2024.

[7] EURORDIS. The voice of 12,000 patients: Experiences and expectations of rare disease patients on diagnosis and care in Europe. https://www.eurordis.org/publications/the-voice-of-12000-patients/. Accessed 16 April 2024.

[8] Lopes MT, Koch VH, Sarrubbi-Junior V, Gallo P, Carneiro-Sampaio M. Difficulties in the diagnosis and treatment of rare diseases according to the perceptions of patients, relatives and health care professionals. *Clinics* (Sao Paulo, Brazil). 2018;73:e68. 10.6061/clinics/2018/e68 PMC5866403 29641803

[9] Zurynski Y, Gonzalez A, Deverell M, Phu A, Leonard H, Christodoulou J et al. Rare disease: a national survey of paediatricians’ experiences and needs. BMJ Paediatrics Open. 2017;1(1). 10.1136/bmjpo-2017-000172 PMC5862166 29637168

[10] von der Lippe C, Diesen PS, Feragen KB. Living with a rare disorder: a systematic review of the qualitative literature. Mol Genet Genomic Med. 2017;5(6):758–73. 29178638 10.1002/mgg3.315 PMC5702559

[11] Pelentsov LJ, Fielder AL, Esterman AJ. The supportive care needs of parents with a child with a Rare Disease: a qualitative descriptive study. J Pediatr Nurs. 2016;31(3):e207–18. 26651231 10.1016/j.pedn.2015.10.022

[12] NEBDS. 2023, Epidermolysis Bullosa: Antenatal Referral and Education https://www.ebdressings.com.au/wp-content/uploads/2021/12/NEDBS-Antenatal-Referral-and-Education-Form.pdf Accessed 16 April 2024.

[13] So JY, Teng J, Epidermolysis Bullosa S. 1998 Oct 7 [Updated 2022 Aug 4]. In: Adam MP, Feldman J, Mirzaa GM, editors. GeneReviews^®^https://www.ncbi.nlm.nih.gov/books/NBK1369/ Accessed 16 April 2024.

[14] Martin K, Geuens S, Asche J, Boden R, Browne F, Downe A, et al. Psychosocial recommendations for the care of children and adults with epidermolysis bullosa and their family: evidence based guidelines. Orphanet J Rare Dis. 2019;14(1):133. 31186066 10.1186/s13023-019-1086-5 PMC6560722

[15] Lucky A, Whalen J, Rowe S, Marathe K, Gorell E. Diagnosis and care of the newborn with Epidermolysis Bullosa. Neoreviews. 2021;e438.G. 10.1542/neo.22-7-e438 34210808

[16] Gonzalez M. Evaluation and treatment of the newborn with epidermolysis bullosa. Semin Perinatol. 2013;37(1):32–9. 23419761 10.1053/j.semperi.2012.11.004

